# Targeting CLL-1 for acute myeloid leukemia therapy

**DOI:** 10.1186/s13045-019-0726-5

**Published:** 2019-04-24

**Authors:** Hongbing Ma, Iyer Swaminathan Padmanabhan, Simrit Parmar, Yuping Gong

**Affiliations:** 1 Hematology Department, West China Hospital, Sichuan University, Chengdu, China; 2 Department of Lymphoma and Myeloma, MD Anderson Cancer Center, Texas University, Houston, USA

**Keywords:** Acute myeloid leukemia, CLL-1, CLEC12A, DCAL-2, hMICL, CD371

## Abstract

Despite major scientific discoveries and novel therapies over the past four decades, the treatment outcomes of acute myeloid leukemia (AML), especially in the adult patient population remain dismal. In the past few years, an increasing number of targets such as CD33, CD123, CLL-1, CD47, CD70, and TIM3, have been developed for immunotherapy of AML. Among them, CLL-1 has attracted the researchers’ attention due to its high expression in AML while being absent in normal hematopoietic stem cell. Accumulating evidence have demonstrated CLL-1 is an ideal target for AML. In this paper, we will review the expression of CLL-1 on normal cells and AML, the value of CLL-1 in diagnosis and follow-up, and targeting CLL-1 therapy-based antibody and chimeric antigen receptor T cell therapy as well as providing an overview of CLL-1 as a target for AML.

## Introduction

Acute myeloid leukemia (AML) is the most common and fatal hematological malignancy in adult patients where the majority have a poor prognosis. Despite major strides in the field of anti-cancer treatments and breakthroughs in immunotherapies, over the past four decades, no significant change in the conventional chemotherapy for AML including induction and consolidation treatment underscores the urgency to develop new methods to improve the prognosis in this deadly disease [1, 2]. Over the past few years, immunotherapy has been recognized as a game changer in the field of hematologic malignancies and solid tumors [3]; however, due to the lack of high specificity of target antigens and heterogeneity of AML, application of a similar strategy to combat AML has been slow overall. For example, targeting a well-recognized surface antigen on AML cells, CD33 can also result in severe pancytopenia due to its high cross-expression on hematopoietic stem cell (HSC). In 2004, Bakker et al. used phage display technology to first identify C-type lectin-like molecule-1 (CLL-1) which is expressed on 92% AML and absent on granulocyte-macrophage progenitors (GMPs) [4]. More importantly, CLL-1 is also expressed on leukemic stem cell (LSC), which possesses the ability to indefinitely self-renew and produce plenty of daughter blast cells with a specific phenotype of CLL-1, CD123, CD44, CD96, CD90, CD32, CD25, and TIM-3, acting as one of most important reasons of leukemia relapse [5–7]. Accordingly, its differential characterizations allow for CLL-1 to be considered as an ideal druggable target for treatment of AML. CLL-1 is also named as c-type lectin domain family 12, member A (CLEC12A), myeloid inhibitory c-type lectin-like receptor (MICL), dendritic cell (DC)-associated C-type lectin 2 (DCAL-2), or CD371 [8–10]. Here, we will review the advance of CLL-1 as a therapeutic strategy for AML.

## The structure and function of CLL-1

C-type lectin-like receptors play a pivotal role in the fight against infection and maintain homeostasis and self-tolerance by recognizing damage-associated—and pathogen-associated—molecular patterns leading to regulation of innate and adaptive immunity [11, 12]. In contrast to classic C-type lectin receptors which are calcium-dependent, C-type lectin-like receptors are calcium-independent due to the absence of residues for calcium binding [13]. Based on the structure, C-type lectin and C-type-like lectin receptors are categorized into type I and type II receptor, where the difference lies on multiple and only one carbohydrate recognition domains in type I and type II, respectively [10]. The cell response depends on the balance between immune receptor tyrosine-based activation motif (ITAM) and immune receptor tyrosine-based inhibitory motif (ITIM) in the intracellular NH2 terminus [14].

**Fig. 1:**
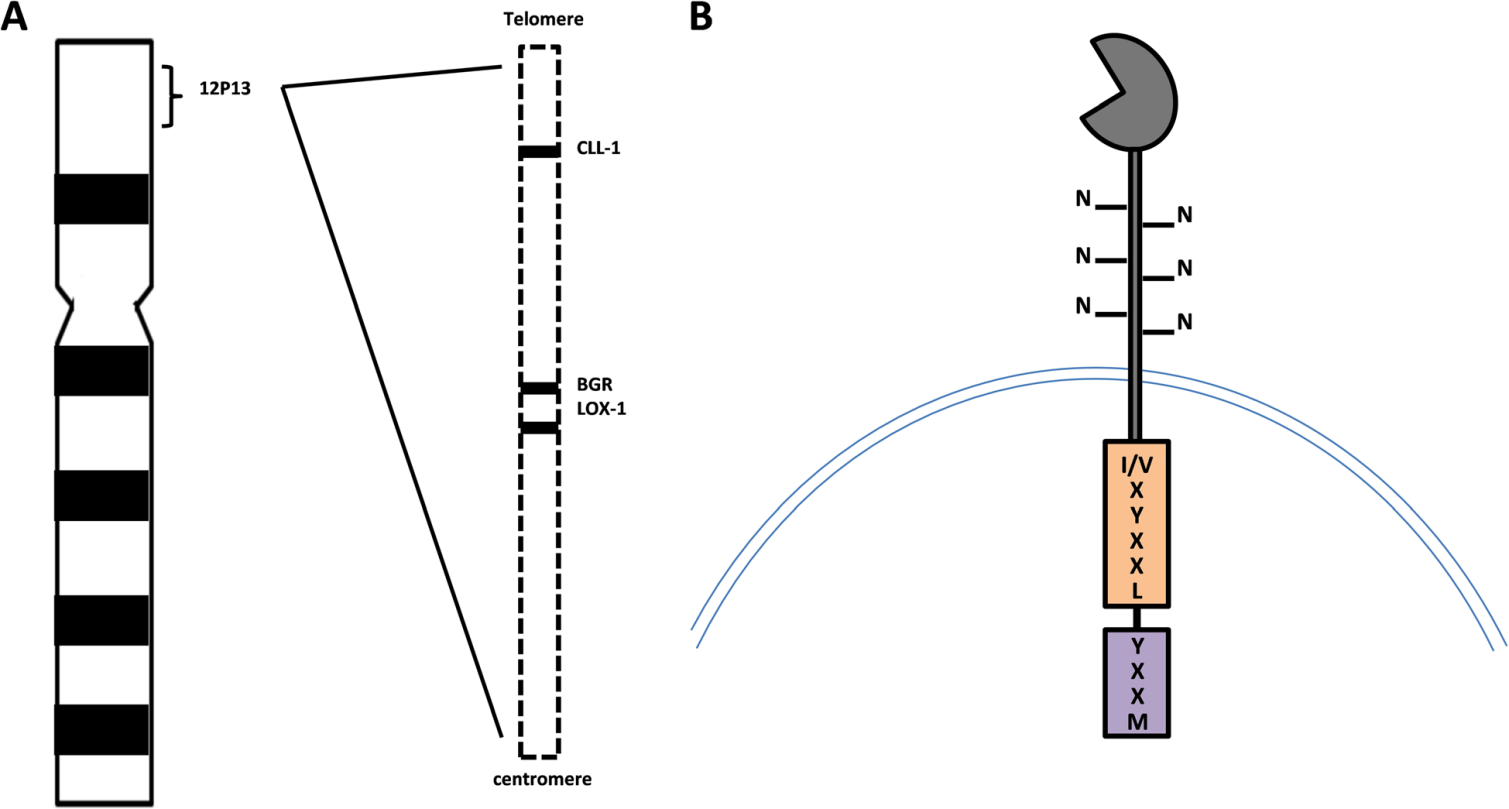
The structure of CLL-1 gene and protein. **a** The human CLL-1 gene maps to 12p13, with high homology to LOX-1 and BGR. **b** CLL-1 protein consists of a single extracellular carbohydrate recognition domains with 6N-glycosylation sites, a transmembrane region, and an intracellular domain with 2 motifs of I/VXYXXL and YXXM at NH2 terminus

CLL-1 belongs to group V of C-type lectin-like receptor family. The human gene encoding CLL-1 maps to 12p13 and is within NK gene complex in which NKG2R, low-density lipoprotein receptor-1 (LOX-1), and β glucan receptor (BGR) are included, CLL-1 is highly homologous to LOX-1 and BGR (Fig. 1a) [4, 13]. The predicted size of CLL-1 gene is about 31 kDa (AY547296), encoding a polypeptide with 275 amino acid [10, 13]. The extracellular feature indicates CLL-1 is a type II transmembrane glycoprotein, consisting of a single extracellular carbohydrate recognition domains with 6 N-glycosylation sites, a transmembrane region and an intracellular NH2 terminus with a sequence of I/VXYXXL and YXXM (Fig. 1b). The I/VXYXXL functions as ITIM, exerting a negative role against cell activation by recruiting inhibitory Src homology region 2 domain-containing phosphatase (SHP)-1 and SHP-2 [4, 13, 15, 16]. The function of YXXM motif in CLL-1 is not clear until now. As YXXM motif carries a binding site for the p85 subunit of phosphatidylinositol 3 kinase (PI3K) which can activate down-stream signals, Darwish et al. speculated the function of CLL-1 may depend on the activation of ITIM or YXXM motif, which possibly decided by the level of phosphorylation of both motifs and the efficiency of recruitment of SHP-1/2 and p85 [15, 17, 18]. Additionally, the YXXM and ITIM motifs have been assumed to be involved in internalization of the CLL-1 receptor upon antibody-mediated cross-linking [19]. The exact role of YXXM in CLL-1 needs to be addressed in the future study.

**Fig. 2:**
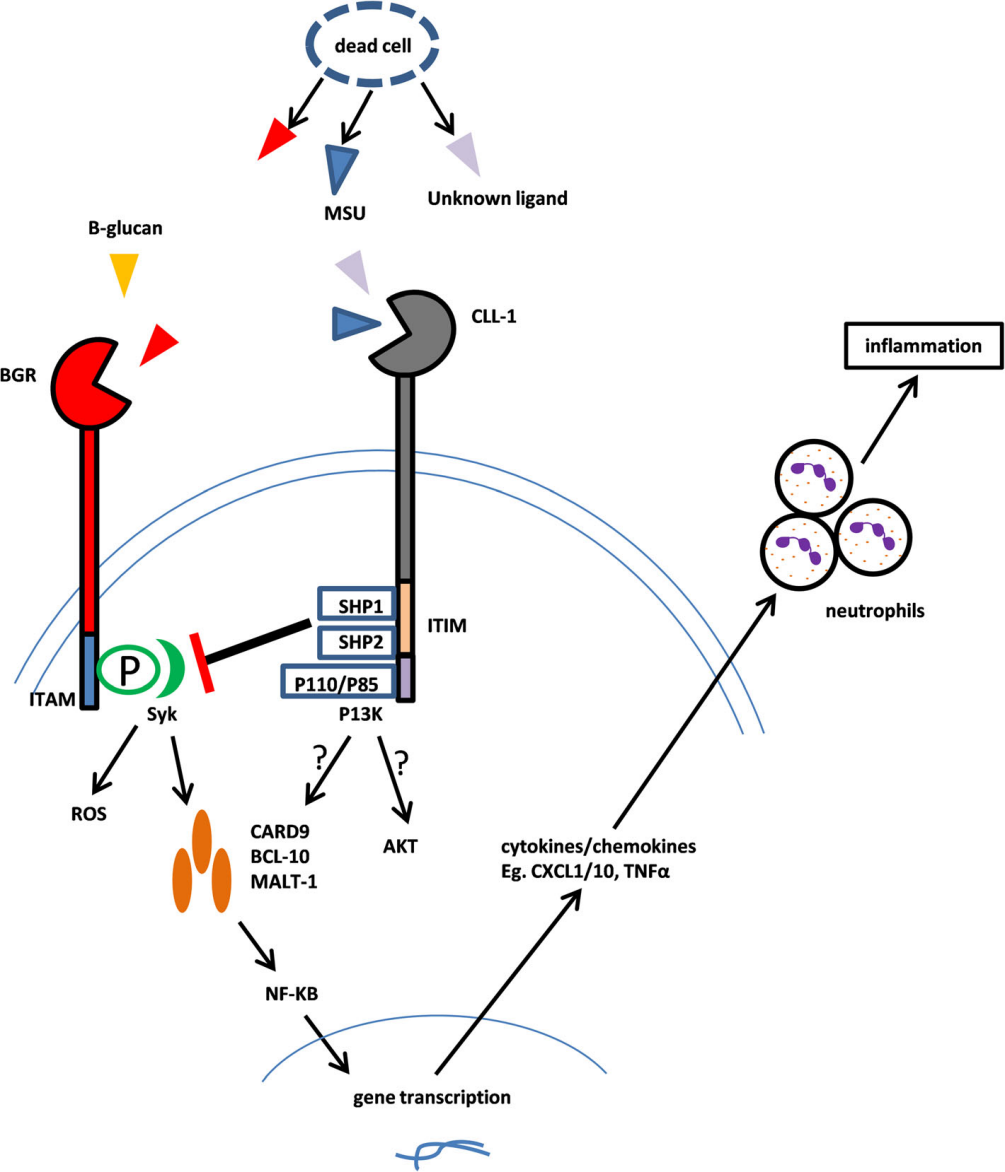
Mechanism of CLL-1 function. The ligands from dead cell or other material trigger the phosphorylation of ITAM and activation of Syk signaling, eliciting the production of reactive oxygen species (ROS) and activation of NF-kB through a complex containing CARD9, MALT1, and Bcl-10; the latter leads to the gene transcription and release of chemokines/cytokines, facilitating neutrophil activation and inflammatory infiltration. This process can be counterbalanced when MSU or unknown ligand on dead cells bind CLL-1, recruiting tyrosine phosphatases SHP-1, and SHP-2 to negatively regulate Syk signaling, as a result, inflammation is eliminated or alleviated. It is unknown whether YXXM in CLL-1 can bind P85 of PI3K and activate the downstream signals

Marshall et al. reported human MICL (hMICL)/CLL-1 was associated with the control of myeloid cell activation during inflammation, it was a negative regulator of granulocyte and monocyte function [9], details in this process depended on the identification of the ligand. Although CLL-1 has a high sequence identity to LOX-1 and BGR which bind apoptotic cells, oxidized low-density lipoprotein and β-glucans, respectively, the exact ligand of CLL-1 is not completely understood [13]. In 2014, Neumann et al. revealed uric acid crystal from dead cell and unknown ligand on dead cells are ligands of CLEC12A/CLL-1, where CLEC12A/CLL-1 plays an essential role in attenuating sterile inflammation which is induced by uric acid crystal in a Syk-dependent pathway. In Clec12a^−/−^ mice, no difference in myeloid cells and lymphoid cells was found from wild-type mice; however, when compared with wild-type mice, monosodium urate (MSU)-induced reactive oxygen species (ROS) specifically and highly increased on both bone marrow cells and purified neutrophils of Clec12a^−/−^ mice. Furthermore, significantly increased neutrophil infiltration occurred in Clec12a^−/−^ mice rather than wild-type mice when MSU crystal or dead cells were injected into peritoneum or total body irradiation of X-ray with a dose to kill double positive thymocytes was given, accompanying with elevated level of CXCL1,CXCL10, and TNF-α (Fig. 2) [12]. Gagne et al. showed MSU could also downregulate expression of MICL in neutrophils, facilitating neutrophil activation and inflammatory infiltration, pretreatment with colchicine could eradicate the downregulating effect [20]. The double-edged sword role of MSU indicates CLL-1 plays a critical role in maintaining homeostasis and that the dysfunction of CLL-1 may elicit autoimmune disease. Some studies have demonstrated CLL-1 may play a role in the development of autoimmune diseases such as rheumatic arthritis and multiple sclerosis [21, 22]. In a collagen antibody-induced arthritis (CAIA) model, Clec12a^−/−^ mice experienced more severe inflammation during CAIA due to the over-activation of myeloid cells [23]; while in multiple sclerosis model, Sagar et al. revealed that CLEC12A/CLL-1 participated in the trafficking of DCs across the blood-brain barrier. The anti-CLEC12/CLL-1 antibody can decrease the DC infiltration within the central nervous system (CNS) while restoring DC function in the peripheral blood which can result in delayed onset of experimental autoimmune encephalomyelitis and alleviation of clinical symptoms as also confirmed in CLEC12A knockout model [21]. Additionally, Begun et al. reported CLEC12A/CLL-1 to be very important in antibacterial defense, where Clec12a^−/−^ mice had a higher risk to be infected due to the impaired antibacterial autophagy [24]. The inconsistent results in these studies may derive from the different target cells, variable disease model and unknown ligands, since the different C-type lectin receptor-ligand partner resulted in variant signals and outcomes [25]. Furthermore, it was also reported that CLL-1 can mediate cell activation by an unclear mechanism [26]. More research on the ligand and pathophysiological mechanism are warranted.

## Expression in normal cells and AML

In mouse model, Pyz et al. revealed the ligand of mouse MICL (mMICL) was widely expressed on bone marrow, thymus, heart, spleen, and kidney, indicating a role in immune hemostasis [27]. However, although there is a similarity in structure and function between mMICL and hMICL, non-hematological tissues in human seldom express CLL-1/hMICL [4, 13]. In the hematopoietic tree, CLL1 is mainly expressed on almost all the granulocytes and monocytes, approx. 61.8% of granulocyte and monocyte precursors; 41.6% of progenitors and only on 2.5% of HSC defined as CD34^+^CD38^−^, but not on T, B, NK cells and erythrocytes and their precursors [28]. CLL1 is also expressed on basophilic, eosinophilic granulocyte, macrophage, and myeloid DC [9, 10, 13, 29]. Among myeloid progenitors defined as CD34^+^CD38^+^CD123^+^CD45RA^+^, Marie Bill et al. reported 39.1% common myeloid progenitors (CMPs), 81% GMPs, and 11.9% megakaryocyte-erythroid progenitors (MEPs) express CLL1 whereas the normal stem cell with lin^−^CD34^+^CD38^−^ does not express CLL1. In fact, the earliest CLL1^+^ cell in normal hematopoiesis is CMPs and CLL1^+^ subsets facilitate colony growth of myelomonocytic lineage [30]. In contrast, CLL1 is present on most primary AML varying from 77.5 to 92% [28, 31]. More importantly, CLL-1 can serve as a marker of the LSC which is regarded as the source of relapse in leukemia. Van et al. reported CLL-1^+^CD34^+^CD38^−^ cells isolated from AML patients can produce leukemia in non-obese diabetic/severe combined immunodeficiency (NOD/SCID) mice, but CLL-1 is negative on the CD34^+^CD38^−^ cells derived from normal bone marrow, regenerating bone marrow after chemotherapy from other diseases and mobilized peripheral blood from non-AML patients [32]. CD123 has not only been regarded as a marker of LSC in AML, but is also highly expressed on non-AML regenerating bone marrow CD34^+^CD38^−^ cells. Therefore, compared with CD123, CLL-1 is a better marker for LSC. With regard to cell line, HL-60, THP-1, and U937 have a high expression of CLL-1 which facilitate to be selected as a target cell for research [4].

## Diagnostic value and follow-up for minimal residual disease

In an analysis of 55 retrospective and 36 prospective samples, Larsen et al. revealed that the expression of hMICL/CLL-1 was restricted to myeloid cells as compared to other stem cell antigens, which indicated CLL-1 can be used as a marker of AML diagnosis [33]. Additionally, CLL-1 expression is stable during the course of the disease and that there is no difference in expression between diagnostic and relapsed samples in the same patients [32, 33]. Eissa et al. compared the phenotype of bone marrow in newly diagnosed AML, in CR, and relapsed patients with ALL and healthy donors, monitoring the change after CR and relapse, they found hMICL/CLL-1 was specifically expressed in AML and showed a stable status during the course of disease [34]. Therefore, CLL-1 can be also used to monitor the minimal residual disease (MRD) of AML in stem cell level during follow-up. Based on the analysis of 397 AML, Coustan-Smith et al. showed that combining CD371/CLL-1 with other 21 markers facilitated distinguishing leukemic cells from normal cells, their stable expression during the disease course made MRD more reliable [35]. Moreover, accumulating evidence demonstrate its predictive value in prognosis. Van et al. reported CLL-1 can be detected and quantified on LSC in patients at diagnosis and in CR after chemotherapy, which makes it ideal to serve as a marker of minimal residual stem cell disease [36]. High LSC frequency at diagnosis is correlated with high MRD frequency after chemotherapy and poor survival [37]. Currently, CLL-1 had been integrated with abnormal makers or other lineage markers to display LSC, showing a negative correlation with survival [36, 38]. As hMICL and CD123 were all markers of LSC, highly and stably expressed in most of AML, Rough et al. reported that combining hMICL/CLL-1 and CD123 with CD45/CD34/CD117 can sensitively detect MRD which is comparable with a real-time quantitative polymerase chain reaction, irrespective of CD34^+^ status [39]. High level of hMICL/CD123 MRD indicated high risk of relapse [33, 39].

Of note, one needs to exclude basophils and part of DCs when CLL-1 is used as a marker to evaluate the MRD, because these cells test positive for CLL-1 and have low CD45^+^ expression and low SSC on flow which is similar to the “blast gate” [30]. Harrington et al. reported basophils consist of averaging 35% of bone marrow cells of myeloproliferative disease when blast gate was used [40]. Therefore, it needs to combine other phenotype markers to differentiate.

## Antibody-based therapy

### Preclinical studies

Bakker et al. reported CLL-1 can efficiently internalize after ligand binding, indicating CLL-1 as target antigen for antibody-based therapy [4]. However, anti-CLL-1 antibody cannot inhibit the proliferation of CLL-1^+^ HL60 cell line, which may indicate such an antibody does not have anti-leukemic effect, the possible reason may be the absence of induction of antibody-dependent cell cytotoxicity (ADCC) and complement-dependent cytotoxicity (CDC). Van et al. proposed to combine a toxic moiety to anti-CLL-1 antibody to induce killing effect [32], where two kinds of anti-CLL-1 antibody-drug conjugate with pyrrolobenzodiazepine (DCLL9718A) and isoquinolidinobenzodiazepine (CLT030), respectively, have shown powerful response to AML in animal models with no or little target off tumor toxicities [41–43]. In contrast, Zhao et al. screened an anti-CLL-1 antibody from a series of candidates which showed ADCC and CDC cytotoxicity against AML cell lines and delayed the progress of HL-60 cells in vivo [19]. The contradictory outcomes may derive from the difference between anti-CLL-1 antibodies. Additionally, based on the fact that tumor necrosis factor-related apoptosis-inducing ligand (TRAIL) can induce or increase the anti-tumor activity of neutrophil and T cell, Wiersma et al. designed a fusion protein scFvCLL1:TRAIL which can equip neutrophils with high density of TRAIL, as a result, the efficacy against AML cell line and other tumor was enhanced and more importantly, the ADCC activity of neutrophils were significantly increased when anti-tumor monoclonal antibody was combined [44]. This provides a novel way to augment the effect of antibody-based therapy. Furthermore, bispecific antibody (T cell-dependent bispecific antibody, TDB) is another strategy. Blinatumomab, a bispecific T cell engager (BiTE) against CD3/CD19, has been approved to treat relapsed and refractory acute lymphoblastic leukemia (ALL), it can redirect and recruit unstimulated primary T cell in patients against CD19-positive malignancy after binding [45]. Utilizing the same strategy, Leong et al. developed an anti-CD3/antiCLL1 T cell-dependent bispecific (TDB) antibody to treat AML and CLL-1 TDB antibody showed potent anti-leukemia activity to AML cell lines. Although high-affinity anti-CD3 TDB antibody demonstrated highly stronger effect than low-affinity anti-CD3 TDB antibody in vitro, they had almost the same effect in mice model. Simultaneously, due to the less cytokine release, low-affinity anti-CD3 TDB antibody was better tolerated than high-affinity anti-CD3 TDB antibody in monkey model, indicating higher safety. Therefore, low-affinity CD3 TDB antibody may be a preferable option for clinical application in the future [1]. Lu et al. also synthesized a bispecific antibody, anti-CLL1-CD3, which showed a superior anti-leukemia activity against AML cell lines and primary AML cells in vitro and in vivo as compared to anti-CD33-CD3 [46]. MCLA-117, a human bispecific IgG antibody which targets CLL-1 and CD3, was generated by Merus B.V. and demonstrated potent cytotoxicity against primary AML cells at a low effector to target ratios in vitro [47]. Related data are summarized in Table 1.

**Table 1:** Preclinical data for anti-CLL-1 antibody-based therapy

Study	Antibody type	In vitro efficacy	Animal model	In vivo efficacy	Adverse effect
Zheng 2019 [41]	Anti-CLL-1-PBD	Highly active against AML cell line and primary AML cells	Mice	Significant decrease in leukemia burden	No weight loss and signs of moribundity
			Cynomolgus monkeys	N/A	Well toleration; welling at injection site; remarkable decrease of granulocyte and monocyte, minimal decrease of RBC; no effect on PLT and lymphocytes
Jiang 2018 [43]	Anti-CLL-1-IQB	Effective to AML cell line and primary AML cell; inhibit LSC colony formation	Mice	Significant decrease in leukemia burden	No effect on engraftment or differentiation of CD34+ cells
Zhao 2010 [19]	Anti-CLL-1 antibody	Effective to AML cell line and primary AML cells	Mice	Delayed tumor growth	N/A
Wiersma 2015 [44]	scFvCLL-1:TRAIL	Upregulating TRAIL on granulocytes, improving anti-tumor activity of granulocyte; enhancing ADCC.	N/A	N/A	N/A
Leong 2017 [1]	Anti-CLL-1/anti–CD3 bispecific antibody	Highly active against CLL-1+ AML cell lines and clinical AML samples, especially for CD3H.	Cynomolgus monkeys	N/A	Vascular shock with CLL-1/CD3H; well toleration with CLL-1/CD3L. Evident decrease of monocyte and granulocyte, early decrease of lymphocyte.
Lu 2014 [46]	Anti-CLL-1/anti–CD3 bispecific antibody	High cytotoxicity to AML cell lines, modest cytotoxicity to primary AML.	Mice	Elimination of the tumor	No effect on body weight and other status
Loo 2015 [47]	Anti-CLL-1/anti–CD3 bispecific antibody	Efficiently activating T cells, potent anti-leukemia against primary AML cells	N/A	N/A	N/A

*PBD* pyrrolobenzodiazepine, *IQB* isoquinolidinobenzodiazepine, *PLT* platelet, *RBC* red blood cell, *PB* peripheral blood, *scFV* single-chain variable fragment, *TRAIL* tumor necrosis factor-related apoptosis-inducing ligand, *ADCC* antibody-dependent cell-mediated cytotoxicity, *CD3H CD3* with high-affinity arm, *CD3L CD3* with low-affinity arm

Compared with other c-type lectin receptors, DACL-2/CLL-1 is mainly expressed on myeloid DC, it can be used as Ag capture receptor due to its internalization after ligand binding and it can also interact with TLR or CD40 to regulate the immune response. Therefore, a strategy of targeting DACL-2/CLL-1 on DCs is also a feasible way for antibody-mediated delivery [10]. Hutten et al. showed CLEC12A/CLL-1 on DCs was an efficient and promising vehicle to present antigen to augment specific CD4^+^ and CD8^+^ T cell immune response against cancer, simultaneously and that the antibody binding did not influence phenotype and function of DCs [8]. However, in contrast to in vitro results, Macri et al. reported in vivo antibody-mediated targeting CLEC12A/CLL-1 on DCs that showed an inferior response to c-type lectin domain family 9 either in cellular immunity or in humoral immunity [48]. Lahound et al. found that DC activation agent could significantly enhance humoral response; moreover, OVA-conjugated with anti-CLEC12A elicited OVA-specific T cell response [49]. The reasons for the difference may derive from different epitope recognition and binding efficiency of antibody or model system; further research is needed to elucidate [8].

### Clinical trial

Up to now, there is only one clinical trial with MCLA-117 which has recruited relapsed, refractory, and newly diagnosed AML in old patients (≥ 65 years) with high-risk cytogenetics or intolerance of induction therapy since 2016. It is a phase 1, multinational and first in a human study with a planned completion time of December 2018, where 50 patients are scheduled to be recruited with the primary goal to determine the maximum tolerated dose and then assesses the safety and efficacy based on recommended dose. The patients receive treatment weekly for 1 cycle, 28 days is 1 cycle, no dose, and any results are released until now (NCT03038230).

## Chimeric antigen receptor T cell therapy

### Preclinical studies

Besides the selective expression on AML blasts and LSC, CLL-1 is also rarely expressed on non-hematological tissues [4, 13], making CLL-1 an ideal target for immunotherapy in AML. Tashiro et al., Eduardo Laborda et al., and Wang et al. developed and optimized CLL-1 CAR-T for AML; they all showed efficient and specific anti-leukemia activity to AML cell lines and primary blasts from AML patients, as well as in mouse model [28, 31, 50]. Concerning the structure of CLL-1 CAR-T, Tashiro et al. found that 4-1BB has the most powerful ability to stimulate T cell to produce specific cytokine and maintain persistent cytotoxicity after comparing one or two combinations of CD28, 4-1BB, and OX40 [31]. It has proved that the length of the space domain also plays a crucial role for anti-leukemia activity. Laborda et al. revealed that the shorter form is better than the longer hinge from human IgG4 in yielding cytokines [50]. In order to avoid continuous activity in vivo, inducible caspase9 suicide gene is designed in the CLL-1 CAR-T cells and can be activated by exogenous drug; a positive effect and efficiency are verified in a mouse model [31]. Kenderian et al. demonstrated that CLEC12A/CLL-1 was overexpressed on AML LSC and that the CLEC12A^+^/CLL-1^+^ AML blasts have a higher risk to be resistant to chemotherapy than their negative counterpart. They generate second CLEC12A CAR-T with 41BB to evaluate the anti-leukemia activity, where the CAR-T cells were highly and specifically effective to CLEC12A cell lines. Although monotherapy with CLEC12A elicited modest anti-leukemia activity, a significant prolonged survival was achieved when it was sequenced after chemotherapy, indicating a preferable option for consolidation to eliminate MRD and LSC [51]. Similar results were also reported in ASH meeting 2018 [52]. Related data are summarized in Table 2.

**Table 2:** Preclinical data of CLL-1 CAR-T cell therapy

Study	Generation	Costimulatory domain	Transduction method	In vitro efficacy	Impact on normal cells	NSG mice model
Tashiro 2017 [31]	Second	4-1BB	Retrovirus	Potent and specific cytotoxicity against CLL-1+ targets	Cytotoxic to mature myeloid cells, sparing myeloid progenitor cells	Significantly prolonged survival
Laborda 2017 [50]	Second	4-1BB	Lentivirus	Robust toxicity on AML cell lines and patient-derived AML blast	Minor decrease in CFU-GM, no impact on BFU-E, CFU-GEMM, and HSC; Neutropenia.	Complete elimination of leukemia by day 90
Kenderian 2016 [51]	Second	4-1BB	Lentivirus	Modest effect to primary AML blasts; highly effective to CLL-1+ AML cell lines.	N/A	100% survival at day 200 in combination with cytarabine while 20% in untreated.
Wang 2018 [28]	Third	CD28 and 4-1BB	Lentivirus	Specific and strong lysis of AML cell line and primary AML blasts	Eradicating mature granulocytes, variable elimination of progenitors, sparing HSC	Significantly decreased leukemia burden and prolonged survival
Togni 2018 [52]	Second	4-1BB	Lentivirus	Dose-dependent killing efficacy	N/A	Significantly prolonged survival with CLL-1 CART-A while not with CART-B

*NSG* NOD/SCID IL2RγCnull, *CFU-GM* granulocyte-macrophage progenitor colonies, *BFU-E* burst-forming units-erythroid, *CFU-GEMM* colony-forming units-granulocyte, erythroid, macrophage, megakaryocyte

### Clinical trials

Bakker et al. reported 67% CD33-AML express CLL1, making CLL-1 a compliment as therapeutic target [4]. On EHA meeting 2018, a team from China reported first-in-human results of a dual target combining CLL1 and CD33, where either antigen of CD33 and CLL-1 can elicit anti-leukemia activity of the compound CART (cCART). As a result, LSC and AML blasts can be eradicated at largest extent by the cCART and in vitro, the cCART showed specific and potent anti-leukemia efficacy against CLL-1 or CD33-positive on both AML cell lines and primary AML cells. In vivo experiments demonstrated the cCART significantly prolonged the survival of the AML mice with U937 or other cell lines. Furthermore, alemtuzumab, acting as a switch, could eliminate the CAR T cells in vivo. Based on the above-mentioned results, the team designed three doses of 1 × 10^6^/kg, 3 × 10^6^/kg, and 9 × 10^6^/kg for escalation in phase I trial. Inspiringly, a 44-year-old male patient with refractory AML (AML-M4, normal karyotype, TP53 mutation) converted to MRD− disease when a dose of 7 × 10^5^/kg CLL-1-CD33 CAR-T cells was firstly used after T cell-depleting conditioning therapy with fludarabine 30 mg/m^2^ and cyclophosphamide 500 mg/m^2^ for 3 consecutive days. Before receiving the CAR-T cell therapy, the patient had refractory disease to 4 cycles of chemotherapy including DA, FLAG, and 2 cycles of priming therapy plus decitabine. The patient tolerated the therapy well and experienced pancytopenia, and only grade 1 cytokine release syndrome (CRS). A matched sibling allogeneic stem cell transplantation was successfully followed, and the patient is alive and disease-free at the time of last follow-up [53]. Recently, on ASH meeting 2018, the same group reported another refractory AML with complex karyotype and FLT3-ITD mutation in a 6-year-old female patient, which was transformed from Fanconi anemia. Followed by the same conditioning therapy, 1 × 10^6^/kg CAR-T cells were used on day 1 and day 2, respectively and a dramatic elimination of AML cells in bone marrow within 1 week, as evidenced by 98% on day 12 and MRD on day 19, accompanying 36% and 60% CAR-T cells in PBMC and bone marrow, respectively, was demonstrated. The patient also experienced pancytopenia and grade 1 CRS, as well as grade 3 neurotoxicity. The patient went on to receive a non-myeloablative hematopoietic cell transplantation where successful hematopoiesis recovery could be seen 2 weeks after HSCT. Unfortunately, the patients succumbed to severe infection [54]. The team is enrolling more patients to accumulate more data, the potent anti-leukemia ability implies this compound CAR-T therapy is more reasonable to act as a bridge to transplantation. Additionally, one phase I/II multi-CAR-T cell therapy targeting Muc1/CLL1/CD33/CD38/CD56/CD123 from China is enrolling patients with refractory or relapsed AML; it plans to enroll 10 patients between 2 and 75 years old and aims to evaluate the feasibility, safety, and efficacy of the fourth generation CAR-T cells, estimated completion date is December 31, 2020. Infusion dose and trial results are not yet available (NCT03222674). Another phase II/III CD123/CLL-1 CAR-T trial from China began to recruit refractory and relapsed AML patients on August 15, 2018; 20 patients younger than 75 years old is scheduled to assess the safety and efficacy, the primary outcome measure is leukemia-free survival of 1 year. Infusion dose is not available and estimated study completion date is August 10, 2021 (NCT03631576). Related data are summarized in Table 3. All the trials enroll relapsed or refractory AML in China.

**Table 3:** Clinical data of CAR-T cell therapy

Study identifier	ICG136	ICG144	NCT03222674	NCT03631576
Clinical phase	I	I	I/II	II/III
Target	CLL-1/CD33	CLL-1/CD33	Muc1/CLL-1/CD33/CD38/CD56/CD123	CD123/CLL-1
Generation	Second	Second	Fourth	N/A
Costimulatory domain	CD28 for CLL-1/4-1BB for CD33	CD28 for CLL-1/4-1BB for CD33	N/A	N/A
Transduction method	Lentivirus	Lentivirus	N/A	N/A
Patient number	1	1	10	20
Age (years)	44	6	2–75	≦75
Conditioning chemotherapy	FC	FC	N/A	N/A
CAR-T dose	7 × 10^5^/kg	1 × 106/kg/days × 2 days	N/A	N/A
Study start	N/A	N/A	2017	2018
Estimated completion date	N/A	N/A	2020	2021
Status	N/A	N/A	Recruiting	Recruiting
Results	MRD− followed by sibling matched HSCT	complete response, followed by Haplo-HSCT	N/A	N/A
Adverse events	Grade 1 CRS, pancytopenia	Grade 1 CRS, grade 3 neurotoxicity, pancytopenia	N/A	N/A

*FC* fludarabine and cyclophosphamide, *CRS* cytokine release syndrome, *FLT-ITD* FMS-like tyrosine kinase-3- internal tandem duplication

## Conclusion

The unique expression pattern makes CLL-1 a preferred target for AML. Although most studies regarding CLL-1 are mainly on anti-CLL-1 antibodies, the first successful utilization in human has inspired more interests in targeting CLL-1 for AML, it can be expected an increasing number of researches on CLL-1 CAR-T will emerge, especially the combination with other markers such as CD123 or CD33 or with novel agents such as FLT3 or BCL-2 inhibitor [2]. Considering the high cost and long time to generate individualized CAR-T cells, universal third-party T cells are developed and utilized to produce a split, universal, and programmable (SUPRA) CAR-T system which demonstrates several superiorities to the previous generations [55]. The efficacy of SUPRA CAR-T CLL-1 is merited to be investigated although there are still many challenges to be addressed in CART therapy [3, 56]. Additionally, due to high expression of CLL-1 on DCs and characteristics of internalization, DCs-based immunotherapy can potentially be exploited as another strategy. At present, more researches are needed to elucidate the exact physiological function of CLL-1; simultaneously, the outcomes from clinical investigations will provide more valuable information.

## Abbreviations

ADCC: Antibody-dependent cell cytotoxicity
ALL: Acute lymphoblastic leukemia
AML: Acute myeloid leukemia
BiTE: Bispecific T cell engager
CAR-T: Chimeric antigen receptor T
cCART: Compound CAR-T
CDC: Complement-dependent cytotoxicity
CLEC12A: c-type lectin domain family 12, member A
CLL-1: C-type lectin-like molecule-1
CMPs: Common myeloid progenitors
CNS: Central nervous system
DC: Dendritic cell
DCAL-2: Dendritic cell-associated C-type lectin 2
GMPs: Granulocyte-macrophage progenitors
hMICL: Human myeloid inhibitory C-type lectin-like receptor
HSC: Hematopoietic stem cell
LSC: Leukemia stem cell
MEPs: Megakaryocyte-erythroid progenitors
mMICL: Mouse inhibitory C-type lectin-like receptor
MRD: Minimal residual disease
NOD/SCID: Non-obese diabetic/severe combined immunodeficiency
PI3K: Phosphatidylinositol 3 kinase
TDB: T cell-dependent bispecific
TRAIL: Tumor necrosis factor-related apoptosis-inducing ligand
